# Supplementary material for: Potentially Inappropriate Prescribing and Potential Prescribing Omissions in 82,935 Older Hospitalised Adults: Association with Hospital Readmission and Mortality within Six Months
Source: Geriatrics (Basel). 2020 Jun 12;5(2):37. doi: 10.3390/geriatrics5020037 (PMC7344435; doi:10.3390/geriatrics5020037)
Supplement: Supplementary file 1 [file geriatrics-05-00037-s001.pdf]

Tables S1-S5. Supplementary Materials

Table S1. Association rules for PIMs or PPOs with readmission or mortality 6 months after discharge.

Rank, by Lift	Itemset	Outcome, readmit (R) or mortality (M)	Count	Support	Confidence	Lift
Frequent AGS PIM sets[a,b]						
1	{AGS2E2,AGS4D,AGS5E}	=> {M}	932	0.011	0.275	4.958
2	{AGS2E2,AGS4D}	=> {M}	1157	0.014	0.217	3.907
3	{AGS2E4,AGS5B}	=> {M}	830	0.010	0.173	3.114
4	{AGS2E4,AGS5E}	=> {M}	838	0.010	0.157	2.820
5	{AGS2E4,AGS4D}	=> {M}	830	0.010	0.136	2.448
6	{AGS2D1,AGS2G1}	=> {R}	894	0.011	0.507	2.239
7	{AGS2G1,AGS4D}	=> {R}	846	0.010	0.494	2.183
8	{AGS2D1,AGS2E2,AGS4D,AGS5E}	=> {R}	878	0.011	0.465	2.054
9	{AGS2G1}	=> {R}	1393	0.017	0.464	2.049
10	{AGS2E4}	=> {M}	961	0.012	0.111	1.997
11	{AGS4D,AGS5E}	=> {M}	1283	0.016	0.111	1.996
12	{AGS2D1,AGS5E}	=> {M}	869	0.011	0.107	1.926
13	{AGS2D1,AGS2E2,AGS4D}	=> {R}	1165	0.014	0.422	1.866
14	{AGS3A1,AGS4D}	=> {R}	1109	0.013	0.420	1.855
15	{AGS2D1,AGS4D,AGS5B,AGS5E}	=> {R}	848	0.010	0.420	1.853
Frequent STOPP PIM sets [c,d]						
1	{STOPPA3,STOPPK2,STOPPL1}	=> {M}	854	0.010	0.487	8.772
2	{STOPPK2,STOPPL1}	=> {M}	899	0.011	0.481	8.666
3	{STOPPA3,STOPPK2}	=> {M}	1026	0.012	0.219	3.947
4	{STOPPK2}	=> {M}	1168	0.014	0.205	3.687
5	{STOPPA3,STOPPK2,STOPPL1}	=> {R}	936	0.011	0.534	2.358
6	{STOPPK2,STOPPL1}	=> {R}	966	0.012	0.517	2.284
7	{STOPPA3,STOPPK1}	=> {M}	873	0.011	0.125	2.250
8	{STOPPA3,STOPPL1}	=> {M}	1554	0.019	0.118	2.116
9	{STOPPA3,STOPPK3,STOPPL1}	=> {M}	1104	0.013	0.111	2.003
10	{STOPPK1}	=> {M}	961	0.012	0.111	1.997
11	{STOPPA3,STOPPD7,STOPPK2}	=> {R}	896	0.011	0.449	1.985

12	{STOPPL1}	=> {M}	1771	0.021	0.107	1.926
13	{STOPPD7}	=> {R}	937	0.011	0.430	1.900
14	{STOPPD7,STOPPK2}	=> {R}	937	0.011	0.430	1.900
15	{STOPPA3,STOPPK1,STOPPK3, STOPPL1}	=> {R}	910	0.011	0.422	1.862
Frequent START PPO sets[e,f]						
1	{STARTA4}	=> {M}	439	0.005	0.198	3.557
2	{STARTA6}	=> {M}	623	0.008	0.132	2.383
3	{STARTF}	=> {R}	525	0.006	0.371	1.639
4	{STARTE4}	=> {M}	467	0.006	0.082	1.471
5	{STARTA8}	=> {R}	639	0.008	0.331	1.464
Frequent START inclusion sets[f,g]						
1	{STARTA5,STARTA6}	=> NOT {M}	1474	0.018	0.985	1.043
2	{STARTA3,STARTA5,STARTA6}	=> NOT {M}	1126	0.014	0.984	1.042
3	{STARTA4,STARTA5,STARTA6}	=> NOT {M}	1038	0.013	0.982	1.040
4	{STARTA3,STARTA5}	=> NOT {M}	1918	0.023	0.978	1.036
5	{STARTA3,STARTA4,STARTA5}	=> NOT {M}	1370	0.017	0.977	1.035

[a] 15 rules with highest lift shown out of 99 rules above support threshold = 0.01

[b] AGS2D1: peripheral a1 blocker; AGS2E2: antipsychotic; AGS2E4: benzodiazepine; AGS2G1: metoclopramide; AGS3A1: thiazolidinedione, NSAID, COX-2 inhibitor… with heart failure; AGS4D: antipsychotic, diuretic, SSRI, SNRI, TCA… (caution); AGS5B: opioid with benzodiazepine; AGS5E: ≥3 CNS-active drugs e.g. antidepressant, benzodiazepine, opioid

[c] 15 rules with highest lift shown out of 185 rules above support threshold = 0.01

[d] STOPPA3: duplicate drug class prescription; STOPPK2: neuroleptic; STOPPL1: oral/transdermal strong opioid; STOPPK1: benzodiazepine; STOPPD7: anticholinergic/antimuscarinic with neuroleptic; STOPPK3: vasodilator with hypotension

[e] 5 rules with highest lift shown out of 15 rules above support threshold = 0.005

[f] STARTA3: antiplatelet with(out) vascular disease; STARTA4: hypertension with(out) antihypertensive; STARTA5: statin with(out) vascular disease; STARTA6: heart failure or CAD with(out) ACE inhibitor; STARTA8: heart failure with(out) appropriate beta-blocker; STARTE4: osteoporosis with(out) bone anti-resorptive; STARTF: diabetes with renal disease with(out) ACE inhibitor or ARB

[g] 5 rules with highest lift shown out of 76 rules above support threshold = 0.01

Supplementary Table S2. Evaluation totals of PIMs and medications to use with caution as recommended by AGS criteria.[1,a]

CODE	Description	Count
Medications to avoid		
Anticholinergics		
2A1	First-generation antihistamine	2708
2A2	Antiparkinsonian agent	85
2A3	Antispasmodic	1325
Antithrombotics		
2B	Dipyridamole, oral short-acting	135
Anti-infective		
2C	Nitrofurantoin	3845
Cardiovascular system		
2D1	Peripheral alpha-1 blocker	36,273
2D2	Central alpha blocker	1970
2D3	Disopyramide	9
2D4	Dronedarone	21
2D5	Digoxin with atrial fibrillation	1174
2D6	Digoxin with heart failure	808
2D7	Digoxin at >0.125 mg/day (searched for: 250mg digoxin)	325
2D8	Nifedipine	2526
2D9	Amiodarone	575
Central nervous system		
2E1	Antidepressant	3690
2E2	Antipsychotic	5332
2E3	Barbiturate	229
2E4	Benzodiazepine	8667
2E5	Meprobamate	0
2E6	Benzodiazepine receptor agonist hypnotic Z drug	13,739
2E7	Ergoloid mesylate	9
Endocrine system		
2F1	Androgen	408
2F2	Desiccated thyroid	0
2F3	Estrogen with or without progestin	2953
2F4	Growth hormone	10

2F5	Insulin, sliding scale	4268
2F6	Megestrol	74
2F7	Sulfonylurea	3214
Gastrointestinal system		
2G1	Metoclopramide unless gastroparesis	3003
2G2	Mineral oil	147
2G3	Proton-pump inhibitor >8 weeks without systemic corticosteroid, NSAID, or esophagitis	10,470
Analgesic drugs		
2H1	Meperidine	43
2H2	Non-COX-2 selective NSAID	9235
2H3	Indomethacin, ketorolac	2820
2H4	Skeletal muscle relaxant	1405
Genitourinary		
2I	Desmopressin	46
PIMs due to exacerbating drug-disease interactions		
Cardiovascular		
3A1	Heart failure with NSAID, COX-2, diltiazem, verapamil, thiazolidinedione, cilostazol, or dronedarone	2999
3A2	Syncope with AChEI, peripheral alpha-1 blocker, tertiary TCA, chlorpromazine…	767
Central nervous system		
3B1	Delirium with anticholinergic, antipsychotic, benzodiazepine…	2714
3B2	Dementia/cognitive impairment with anticholinergic, benzodiazepine, H2-receptor antagonist…	2195
3B3	Falls/fractures with anticonvulsant, antipsychotic, benzodiazepine…	6663
3B4	Parkinson with antipsychotic or antiemetic	64
Gastrointestinal		
3C	Gastric/duodenal ulcer with >325 mg/day aspirin or non-COX-2 selective NSAID	383
Kidney/urinary tract		
3D1	CKD Stage IV or less with NSAID	0
3D2	Urinary incontinence with oral/transdermal estrogen, peripheral alpha-1 blocker (women)	3153

3D3	Lower urinary tract symptoms, prostatic hyperplasia with strongly anticholinergic drug (men)	108
Medications to be used with caution		
4A	Aspirin at age ≥70 for primary prevention of CVD or colorectal cancer	3502
4B	Dabigatran at age ≥75	880
4C	Prasugrel at age ≥75	3
4D	Antipsychotic, diuretic, SSRI, SNRI, TCA…	40,806
4E	Dextromethorphan/quinidine	112
4F	Trimethoprim-sulfamethoxazole	0
Drug-drug interactions to avoid		
5A	RAS inhibitor, amiloride, or triamterene with another RAS inhibitor and CKD stage 3a or higher	329
5B	Opioid with benzodiazepine	4800
5C	Opioid with gabapentin or pregabalin	4874
5D	≥2 anticholinergics	4558
5E	≥3 CNS-active drugs (TCA, SSRI, SNRI, antipsychotic, antiepileptic, benzodiazepine, nonbenzodiazepine, hypnotic Z-drug, opioid)	14,306
5F	Systemic corticosteroid with NSAID	4482
5G	Lithium with ACE inhibitor	39
5H	Lithium with loop diuretic	25
5I	Peripheral alpha-1 blocker with loop diuretic	3751
5J	Phenytoin with trimethoprim-sulfamethoxazole	0
5K	Theophylline with cimetidine	0
5L	Theophylline with ciprofloxacin	6
5M	Warfarin with amiodarone	572
5N	Warfarin with ciprofloxacin	1138
5N	Warfarin with macrolide (exc. Azithromycin)	442
5O	Warfarin with trimethoprim-sulfamethoxazole	1
5Q	Warfarin with NSAID	2811

[a] 21 medications to be avoided or reduce dosage with impaired kidney function not evaluated due to missing creatinine clearance data (Table 6 in [1]).

Supplementary Table S3. Evaluation totals of PIMs as recommended by STOPP criteria.[2]

CODE	Description	Count
	Indication of medication	
A1	Any drug prescribed without evidence-based clinical indication	N/A [a]
A2	Any drug prescribed beyond the recommended duration	N/A [a]
A3	Any duplicate drug class prescription, e.g. two concurrent NSAIDs, SSRIs, SNRIs, loop diuretics, ACE inhibitors, anticoagulants	49,949
	Cardiovascular system	
B1	Digoxin for heart failure with normal systolic ventricular function	808
B2	Diltiazem or verapamil with NYHA Class III or IV heart failure	386
B3	Beta-blocker in combination with diltiazem or verapamil	1013
B4	Beta-blocker with bradycardia, type II or complete heart block	645
B5	Amiodarone as first-line antiarrhythmic therapy in supraventricular tachyarrhythmias	19[b]
B6	Loop diuretic as first-line treatment for hypertension	7431
B7	Loop diuretic for dependent ankle oedema without evidence	39
B8	Thiazide diuretic with significant hypokalaemia, hyponatraemia, hypercalcaemia, or history of gout	1156
B9	Loop diuretic for treatment of hypertension with concurrent urinary incontinence	70
B10	Centrally-acting antihypertensive unless clear intolerance of, or lack of efficacy with, other classes of antihypertensives	1970[b]
B11	ACE inhibitor or ARB with hyperkalaemia	415
B12	Aldosterone antagonist with concurrent potassium-conserving drugs without monitoring of serum potassium	2756[b]
B13	PDE5 inhibitor in severe heart failure characterised by hypotension or concurrent nitrate therapy for angina	212
	Antiplatelet/anticoagulant drugs	
C1	Long-term aspirin at >160 mg/day [d]	51[c]
C2	Aspirin with a history of peptic ulcer disease without concomitant PPI	75
C3	Aspirin, clopidogrel, dipyridamole, vitK antagonist, or thrombin/factor Xa inhibitor with concurrent bleeding risk	17,350
C4	Aspirin plus clopidogrel as secondary stroke prevention, unless stent	745

C5	Aspirin with vitK antagonist or thrombin/factor Xa inhibitor with chronic atrial fibrillation	1461
C6	Antiplatelet with vitK antagonist, thrombin/factor Xa inhibitor with stable coronary, CV or peripheral arterial disease	500
C7	Ticlopidine	6
C8	VitK antagonist or thrombin/factor Xa inhibitor for first DVT	236[b]
C9	VitK antagonist or thrombin/factor Xa inhibitor for first pulmonary embolus	917[b]
C10	NSAID with vitK antagonist or thrombin/factor Xa inhibitor	5298
C11	NSAID with concurrent antiplatelet without PPI prophylaxis	7366
Central nervous system and psychotropic drugs		
D1	TCA with dementia, narrow angle glaucoma, cardiac conduction abnormalities, prostatism, or urinary retention	229
D2	Initiation of TCA as first-line antidepressant treatment	146[b]
D3	Neuroleptic with antimuscarinic/anticholinergic effects with history of prostatism or urinary retention	176
D4	SSRI with recent hyponatraemia	286
D5	Benzodiazepine for ≥4 weeks	5579
D6	Antipsychotic with Parkinsonism or Lewy Body disease	66
D7	Anticholinergic/antimuscarinic to treat extra-pyramidal side effects of neuroleptic	2178
D8	Anticholinergic/antimuscarinic with delirium or dementia	2068
D9	Neuroleptic antipsychotic in patients with behavioural and psychological symptoms of dementia unless severe and after failed treatments	1894[b]
D10	Neuroleptic as hypnotic, unless psychosis or dementia	37
D11	ACE inhibitor with a history of persistent bradycardia, heart block, recurrent syncope or concurrent drugs that reduce heart rate	715
D12	Phenothiazine as first-line treatment	400[b]
D13	Levodopa or dopamine agonist for benign essential tremor	16
D14	First-generation antihistamine	16
Renal system[d]		
E1	>125 µg/day digoxin if eGFR < 30 mL/min/1.73m^2	0
E2	Direct thrombin inhibitor if eGFR < 30 mL/min/1.73m^2	73
E3	Factor Xa inhibitor if eGFR < 15 mL/min/1.73m^2	240
E4	NSAID if eGFR < 50 mL/min/1.73m^2	5731

E5	Colchicine if eGFR < 10 mL/min/1.73m^2	29
E6	Metformin if eGFR < 30 mL/min/1.73m^2	923
Gastrointestinal system		
F1	Prochlorperazine/metoclopramide with Parkinsonism	0
F2	PPI at full therapeutic dosage for > 8 weeks	1604
F3	Antimuscarinic/anticholinergic, oral iron, opioid, verapamil, or aluminium antacid with chronic constipation	546
F4	>200 mg/day oral elemental iron	0
Respiratory system		
G1	Theophylline for COPD	30
G2	Systemic corticosteroid with moderate-severe COPD	1909
G3	Anti-muscarinic bronchodilator with narrow angle glaucoma or bladder outflow obstruction	73
G4	Benzodiazepine with acute or chronic respiratory failure	122
Musculoskeletal system		
H1	Non- COX-2 selective NSAID with peptic ulcer disease or gastrointestinal bleeding, unless with PPI or H2 antagonist	840
H2	NSAID with severe hypertension	13,630
H3	>3 months NSAID for osteoarthritis pain relief where acetaminophen has not been tried	20[b]
H4	>3 months systemic corticosteroid for rheumatoid arthritis	2
H5	Systemic corticosteroid for osteoarthritis	623
H6	>3 months NSAID or colchicine for gout with no contraindication to xanthine-oxidase inhibitor	17
H7	COX-2 selective NSAID with cardiovascular disease	116
H8	NSAID with systemic corticosteroid without PPI prophylaxis	1596
H9	Oral bisphosphonate with upper gastrointestinal disease or bleeding, or peptic ulcer disease	373
Urogenital system		
I1	Antimuscarinic with dementia, chronic cognitive impairment, narrow-angle glaucoma, or prostatism	2092
I2	Alpha-1 alpha blocker with orthostatic hypotension or micturition syncope	1609
Endocrine system		
J1	Sulphonylurea with long duration of action	2736

J2	Thiazolidenedione with heart failure	9
J3	Beta-blocker in DM with frequent hypoglycaemia	8637
J4	Oestrogen with breast cancer history or venous thromboembolism	89
J5	Oral oestrogen without progestogen with intact uterus	435
J6	Androgen without hypogonadism	409
Fall risk		
K1	Benzodiazepine	8667
K2	Neuroleptic	5704
K3	Vasodilator with persistent postural hypotension	56,396
K4	Hypnotic Z-drug	13,739
Analgesic drugs		
L1	Oral or transdermal strong opioid as first line therapy for mild pain	16,556[b]
L2	Regular opioid without concomitant laxative	25,880[e]
L3	Long-acting opioid without short-acting opioid for break-through pain	4433
Antimuscarinic/anticholinergic drug burden		
M	Concomitant use of ≥2 antimuscarinic/anticholinergic drugs	4558

[a] Cannot verify

[b] Cannot fully verify treatment/condition history

[c] Searched for ASA 325mg

[d] Dependent on available eGFR results. Additional possible counts due to missing eGFR data were: 37 (E1), 163 (E2), 847 (E3), 3554 (E4), 183 (E5), 1633 (E6)

[e] Cannot distinguish regular opioid from *"pro re nata"* (PRN) opioid

Supplementary Table S4. Evaluation totals of PPOs and prescribing inclusions as recommended by START criteria.[2]

CODE	Description	Omission count	Inclusion count
Cardiovascular system			
A1	VitK antagonist or thrombin/factor Xa inhibitor with chronic atrial fibrillation	1528[a]	6348
A2	Aspirin with chronic arial fibrillation and contraindicated VitK antagonist or thrombin/factor Xa inhibitor	1528[a]	934
A3	Antiplatelet with coronary, cerebral or peripheral vascular disease	1301	2584
A4	Antihypertensive therapy for hypertension	2222	30,606
A5	Statin with coronary, cerebral or peripheral vascular disease	529	2673
A6	ACE inhibitor with systolic heart failure or coronary artery disease	4708	4570
A7	Beta-blocker with ischaemic heart disease	163	687
A8	Appropriate beta-blocker with stable systolic heart failure	1928	4441
Respiratory system			
B1	Inhaled beta-2 agonist or antimuscarinic bronchodilator for mild/moderate asthma or COPD	1521	5175
B2	Inhaled corticosteroid for moderate-severe asthma or COPD	2263	4433
B3	Home continuous oxygen with chronic hypoxaemia	N/A [b]	
Central nervous system and eyes			
C1	L-DOPA or dopamine agonist in Parkinson's with functional impairment/disability	299	627
C2	Non-TCA antidepressant with persistent major depressive symptoms	562	1249
C3	ACE inhibitor for mild-moderate Alzheimer's or Lewy Body dementia	704	314
C4	Topical prostaglandin, prostamide or beta-blocker for primary open-angle glaucoma	4	13
C5	SSRI, SNRI or pregabalin for persistent severe anxiety	555	592
C6	Dopamine agonist for Restless Legs Syndrome without iron deficiency and severe renal failure	36	34

Gastrointestinal system				
D1	PPI with severe gastro-oesophageal reflux disease or peptic stricture	366		1667
D2	Fibre supplement for diverticulosis with constipation		N/A [c]	
Musculoskeletal system				
E1	Disease-modifying anti-rheumatic drug with active, disabling rheumatoid disease	424		411
E2	Bisphosphonates, vit D and calcium with long-term systemic corticosteroid		N/A [c]	
E3	Vit D and calcium with osteoporosis or fracture		N/A [c]	
E4	Bone anti-resorptive or anabolic therapy with osteoporosis	5718		2353
E5	Vit D with falls or osteopenia		N/A [c]	
E6	Xanthine-oxidase inhibitor with recurrent gout	547		687
E7	Folic acid supplement with methotrexate	282		806
Endocrine system				
F	ACE inhibitor or ARB in diabetes with renal disease	1415		2674
Urogenital system				
G1	Alpha-1 receptor blocker with prostatism and no prostatectomy	998		1190
G2	5-alpha reductase inhibitor with prostatism and no prostatectomy	1896		292
G3	Topical/pessary oestrogen for atrophic vaginitis	6		6
Analgesic drugs				
H1	High-potency opioid in moderate-severe pain, where paracetamol, NSAIDs or low-potency opioids are inappropriate or ineffective	2418		3609 [d]
H2	Laxative with regular opioids	25,471[c]		7053
Vaccines				
I1	Annual seasonal trivalent influenza vaccine		N/A [b]	
I2	Pneumococcal vaccine at least once after age 65		N/A [b]	

[a] Overlapping omissions
[b] Cannot verify
[c] Cannot fully verify OTC fibre, vitamin D, calcium or laxatives
[d] Cannot fully verify condition

Supplementary Table S5. 2016 Age distribution of ≥ 65 year old populations for patients admitted to Calgary hospitals and overall populations of Calgary, Canada and the US.

Age group	Patients admitted to Calgary hospitals[a] (%)	Calgary (%)[3]	Canada (%)[3]	US (%)[4]
65-69	23.5	36.0	33.2	34.2
70-74	19.2	23.0	23.9	24.0
75-79	18.6	16.3	17.2	17.0
80-84	17.8	12.2	12.6	11.9
85-89	12.9	8.0	8.1	7.9
90+	7.9	4.4	4.9	5.1
Total population	25,725	138,405	5,935,630	49,244,195

[a] Distribution is for admissions in 2016, which facilitates direct comparison with the census population distributions.

REFERENCES

1. By the American Geriatrics Society Beers Criteria Update Expert P. American Geriatrics Society 2019 Updated AGS Beers Criteria(R) for Potentially Inappropriate Medication Use in Older Adults. *J Am Geriatr Soc.* 2019;67(4):674-694.
2. O'Mahony D, O'Sullivan D, Byrne S, O'Connor MN, Ryan C, Gallagher P. STOPP/START criteria for potentially inappropriate prescribing in older people: version 2. *Age Ageing.* 2015;44(2):213-218.
3. Statistics Canada. Census Profile, 2016 Census. 2019; www12.statcan.gc.ca/census-recensement/2016/dp-pd/prof/index.cfm?Lang=E. Accessed 28/05/19.
4. United States Census Bureau. National Population by Characteristics: 2010-2017. 2018; www.census.gov/data/datasets/2017/demo/popest/nation-detail.html. Accessed 25/04/19.
